# Understanding vaccine hesitancy in Slovakia: mapping slovak population through latent class analysis

**DOI:** 10.1080/21642850.2026.2667618

**Published:** 2026-05-03

**Authors:** Alexandra Šurinová, Veronika Korim, Veronika Jurková, Radomír Masaryk

**Affiliations:** a Institute of Applied Psychology, Faculty of Social and Economic Sciences, Comenius University, Bratislava, Slovakia; b Institute of Computer Science, Institute of Mathematics, Faculty of Science, Pavol Jozef Šafárik University, Košice, Slovakia; c Department of Psychology, Faculty of Arts, Comenius University Bratislava, Slovakia

**Keywords:** vaccine hesitancy, health literacy, latent class analysis, vaccination attitudes, Slovakia

## Abstract

**Background:**

Vaccine hesitancy and public adherence to health measures remain critical challenges in Slovakia, where coverage of COVID-19 vaccination has been low, and parents’ decisions regarding vaccination of their children further illustrate variability in immunization uptake.

**Methods:**

This study investigates how Slovak adults (*N* = 505) differ in their perceptions of vaccination, compliance with restrictions, and health literacy. Using Latent Class Analysis, we identified three distinct population segments that reflect both internationally recognized typologies and the unique sociocultural context of Slovakia.

**Results:**

Our findings indicate that health literacy is an important characteristic distinguishing population segments with different vaccine-related beliefs and behaviours. Individuals with higher health literacy demonstrated greater confidence in vaccination and more autonomous, informed decision-making, whereas those with lower literacy relied more on external authority and restrictive measures.

**Conclusions:**

These results highlight the importance of tailored public health communication strategies that prioritize clarity, accessibility, and credibility, alongside interventions that combine education and community engagement. By targeting health literacy, policymakers can potentially reduce vaccine hesitancy and foster sustainable trust in health systems. Future research should explore longitudinal and mixed-method approaches to examine causal mechanisms and the dynamic interplay of literacy, trust, and social influence over time.

## Introduction

Vaccination represents one of the most effective preventive measures in combating life-threatening diseases. Each year, it contributes to saving millions of lives by stimulating the body’s natural defence mechanisms and facilitating the development of immune protection (World Health Organisation, n.d.). However, Slovakia ranks among the countries where public interest in and trust toward vaccination have been steadily declining. During the COVID-19 pandemic, Slovakia was among the European countries with the lowest vaccination rates (European Centre for Disease Prevention & Control, 2023a). This downward trend has affected both mandatory and voluntary vaccination programs: by 2024, the national coverage rate for the MMR vaccine had fallen below the threshold required to ensure herd immunity, while uptake of voluntary adult immunisations, including COVID-19 and influenza vaccination, has remained persistently low. At the same time, Slovakia is a bottom-ranking country with regard to influenza vaccination, which is voluntary and targets adults, highlighting ongoing challenges in promoting vaccine acceptance among the adult population (Úrad verejného zdravotníctva SR, 2024a). In this respect, Slovakia represents an analytically relevant context for examining how vaccination attitudes emerge in societies characterised by relatively low levels of institutional trust and persistent public scepticism toward state institutions. Studying vaccination attitudes in such a context may therefore contribute not only to understanding the Slovak case itself but also to broader discussions on vaccine hesitancy and public health communication in low-trust democratic settings.

Multiple factors have been identified as influencing vaccine acceptance, including (mis)trust in physicians and healthcare institutions (e.g. Masaryk, 2016; Murphy et al., 2021; Raffetti et al., 2022; Roy et al., 2022; Zvoníček, 2019), religious beliefs (Murphy et al., 2021; Troiano & Nardi, 2021), education level (Fisher et al., 2020; Troiano & Nardi, 2021; Veldwijk et al., 2015), belief in conspiracy theories (Murphy et al., 2021; Roy et al., 2022), the influence of social reference groups (Masaryk, 2016; Roy et al., 2022). Among these determinants, health literacy emerges as a particularly critical factor, as it shapes individuals’ capacity to access, comprehend, and critically evaluate health information, thereby influencing their ability to make informed vaccination decisions (Johri et al., 2015; Veldwijk et al., 2015). Taken together, these findings underscore that vaccine acceptance is a multidimensional phenomenon shaped by both cognitive and social mechanisms. Among these, health literacy constitutes a crucial cognitive determinant, influencing how individuals interpret medical information, assess risks, and form vaccination attitudes.

Recent person-oriented research further suggests that vaccine hesitancy should not be conceptualised as a simple continuum ranging from acceptance to refusal, but rather as a heterogeneous construct composed of distinct psychological profiles. Latent profile analyses have identified differentiated configurations of hesitancy characterised by varying combinations of institutional trust, reliance on personal information sources, endorsement of conspiracy beliefs, and orientation toward alternative health practices (e.g. Lamot et al., 2024). These findings indicate that distrust, dissatisfaction, and conspiratorial thinking tend to cluster into distinct patterns rather than forming a single underlying dimension of hesitancy. This perspective underscores the importance of segmentation approaches, such as Latent Class Analysis, for capturing the multidimensional structure of vaccination attitudes.

Health literacy can be defined as an individual’s ability not only to access but also to comprehend and appropriately interpret essential health information. This competency is crucial for making informed decisions regarding one’s own health (Parker & Ratzan, 2010). Therefore, to make an informed decision about vaccination, individuals must be able not only to passively receive information but also to critically evaluate and apply it within a specific decision-making context.

A suitable theoretical framework for understanding differences in vaccination decision-making is Ajzen (1991). According to this theory, individual behaviour is guided by three primary determinants: attitudes toward the behaviour, subjective norms, and perceived behavioural control. Attitudes reflect the individual’s evaluation of vaccination as beneficial or harmful, subjective norms represent perceived social pressure from family, peers, or society to get vaccinated, and perceived behavioural control captures the individual’s sense of ability or autonomy to act upon that intention (Ajzen, 1991).

In the context of vaccination, these components interact to shape whether an individual decides to receive a vaccine. Positive attitudes toward vaccination, trust in scientific and institutional sources, and supportive social norms generally increase the likelihood of vaccination, while scepticism, misinformation, or social resistance may weaken behavioural intentions. Similarly, limited perceived control—such as difficulties in accessing vaccines or doubts about personal competence to assess medical information—can further reduce vaccination uptake.

Within this framework, health literacy may function as an important background factor shaping how individuals form attitudes toward vaccination, interpret social expectations, and evaluate their own ability to understand and use health-related information. Individuals with higher health literacy may be better equipped to process complex medical information, critically evaluate competing claims, and develop more informed attitudes toward vaccination. At the same time, higher literacy may strengthen perceived behavioural control by increasing individuals’ confidence in their ability to navigate health information and make autonomous health decisions.

The Theory of Planned Behaviour (Ajzen, 1991) thus provides a comprehensive explanatory model for understanding the cognitive, social, and contextual mechanisms that underlie vaccination decisions. In this sense, health literacy can be understood as a cognitive resource that may shape several components of the TPB simultaneously, particularly attitudes toward vaccination and perceived behavioural control. It helps to clarify why some individuals fully endorse vaccination as a rational and socially responsible act, while others remain hesitant or reject it due to conflicting beliefs, perceived social pressures, or feelings of limited control.

Building on a similar conceptual foundation, Thaker (2021a) conducted a national survey in New Zealand (*N* = 1 040) using Latent Class Analysis (LCA) to identify four distinct classes based on attitudes toward vaccination: enthusiasts, supporters, hesitants, and sceptics.

Vaccine enthusiasts were mostly older adults (56 years and above) with higher education. Over 80% reported never refusing vaccination, and most indicated they would vaccinate their children. They supported restrictions for the unvaccinated (e.g. travel limitations or surcharges) and showed high trust in scientists and mass media as primary information sources.

Vaccine supporters generally agreed with vaccination (74%), were aged between 18 and 45 years, and were highly educated. Like the enthusiasts, they typically did not refuse vaccines before the pandemic, approved of child vaccination, and supported restrictions for the unvaccinated. Compared with other groups, they expressed somewhat greater trust in social media while maintaining high trust in scientists and mass media.

The hesitant group, characterised by lower or medium education levels and ages between 18 and 45 years, represented an undecided population segment that could be influenced in either direction. About 53% stated they would get vaccinated, while the remainder would not. Roughly 15% had previously refused vaccination, around 60% would vaccinate their children, and 35% were undecided. They rarely supported restrictions for the unvaccinated and primarily relied on social media as their main information source.

In contrast, vaccine sceptics expressed strong opposition to vaccination—99% reported they would not get vaccinated. This group consisted mainly of older adults (46 years and above) with lower education levels. One-third did not state their opinion on child vaccination, and 18% admitted to refusing mandatory vaccines in the past. Sceptics rejected restrictions for the unvaccinated and were the only group reporting trust primarily in personal acquaintances, while distrusting scientists and other institutional sources.

In a subsequent Australian study, Thaker et al. (2023) replicated these four classes and identified a fifth one—vaccine socials. Members of this class were undecided but more likely to get vaccinated once vaccines became available. Their motivations centred on self-protection, protecting others, and a desire to return to normal life. Conversely, those who ultimately refused vaccination cited concerns about side effects and the rapid pace of vaccine development.

Such segmentation of the population according to vaccine attitudes provides valuable insight into the spectrum of public opinion and informs the design of more targeted and effective vaccination communication and intervention strategies. In the case of Slovakia—where the COVID-19 vaccination rate reached only slightly above 50% of the population and more than 20,000 people died from the disease—examining vaccination attitudes is particularly crucial. The present study therefore aimed to identify the proportions of vaccine enthusiasts, supporters, hesitants, and sceptics within the Slovak population, and to examine their levels of health literacy, a key factor influencing vaccination-related decision-making (Johri et al., 2015; Veldwijk et al., 2015).

Building on this theoretical framework and previous empirical findings, the present study applied Latent Class Analysis (LCA) to explore whether similar patterns of vaccination-related attitudes and behaviours identified by Thaker (2021a, 2023) in New Zealand and Australia would also emerge within a smaller European context. Specifically, we aimed to identify distinct subgroups within the Slovak population based on their attitudes toward COVID-19 vaccination and to examine how these groups relate to levels of health literacy and other behavioural and perceptual variables, highlighting that the present study represents a contextual adaptation of Thaker’s typology rather than a direct replication, while also providing insight into how vaccination attitudes may be structured in European contexts characterised by relatively low institutional trust.

## Methods

To address the research objectives, we conducted a cross-sectional quantitative survey focusing on attitudes toward COVID-19 vaccination and health literacy in the Slovak adult population. The study design followed the approach established by Thaker (2021a, 2023), employing Latent Class Analysis (LCA) to identify subgroups based on respondents’ vaccination-related beliefs and behaviours. In addition to vaccination attitudes, the survey measured health literacy across nine dimensions and included relevant sociodemographic variables, as well as behavioural and perceptual variables related to COVID-19 vaccination, such as vaccination status, vaccination intentions, perceived vaccine safety and effectiveness, and trust in vaccination.

### Sample

The sample was designed to be representative of the adult population of Slovakia (aged 18 years and older), ensuring proportionality with respect to age, gender, and geographic distribution through a proportional random sampling procedure. The study included a total of 505 respondents, comprising 48.3% men (*N* = 244) and 51.7% women (*N* = 261), with a mean age of 45.2 years (SD = 16.2). Participants were drawn from all regions of Slovakia, which were approximately evenly represented: Bratislava 11.5%, Trnava 10.5%, Trenčín 11.3%, Nitra 13.3%, Žilina 12.9%, Banská Bystrica 12.7%, Prešov 14.1%, and Košice 13.9%.

Regarding educational attainment, 2.4% of participants had completed primary education, 54.1% secondary education, 7.3% held a bachelor’s degree, 32.9% a master’s degree, and 3.4% had completed doctoral studies. In terms of employment status, 52.9% of respondents were employed, 6.1% self-employed, 3.6% unemployed, 6.7% students, 3.6% were individuals with significant health limitations or recipients of disability pensions, 20.5% were retired, and 6.7% were engaged in household or caregiving responsibilities.

### Data collection

The questionnaire consisted of two parts: the Health Literacy Questionnaire (HLQ; Osborne et al., 2013), which measures health literacy using 44 items across nine dimensions, and a set of 21 items originally used in a public opinion survey in New Zealand (Thaker & Cook, 2022). For the HLQ, we employed the validated Slovak version (Kolarcik et al., 2017). Consistent with the original instrument, the Slovak version comprises nine dimensions: (1) feeling understood and supported by healthcare providers (HPS), (2) having sufficient information to manage one’s health (HSI), (3) actively managing health (AMH), (4) social support for health (SS), (5) appraisal of health information (CA), (6) ability to actively engage with healthcare providers (AE), (7) navigating the healthcare system (NHS), (8) ability to find good health information (FHI), and (9) understanding health information well enough to know what to do (UHI). Each dimension includes 4–6 items. Dimensions 1–5 are measured using a four-point agreement scale (strongly disagree to strongly agree), while dimensions 6–9 use a five-point scale assessing the perceived difficulty of health-related tasks (cannot do/always difficult to always easy). Scores for each dimension are calculated as the mean of the corresponding items, with higher scores indicating higher levels of health literacy in the respective domain (range 1–4 for dimensions 1–5 and 1–5 for dimensions 6–9). The psychometric evaluation of the Slovak version confirmed the robustness of all nine dimensions and demonstrated satisfactory reliability, with Cronbach’s *α* ranging from 0.73 to 0.82 (Kolarcik et al., 2017).

The questionnaire by Thaker and Cook (2022) comprises 21 items addressing vaccination intentions, perceptions, and attitudes (Cronbach’s *α* = 0.92) and includes several response formats, such as five-point Likert scales (e.g. strongly agree to strongly disagree), seven-point semantic differential scales (e.g. bad–good, unpleasant–pleasant, undesirable–desirable), yes/no questions, multiple-choice items, and several open-ended responses. The questionnaire was translated into Slovak prior to data collection using the back-translation method. Initially, the items were translated into Slovak by the study authors, and subsequently back-translated into English by a native speaker. To ensure contextual relevance, certain items were adapted to reflect the contemporary situation—for instance, the original question ‘Would you get vaccinated when the vaccine becomes available?’ was reformulated as ‘Are you vaccinated?’. The translated version was also reviewed by the research team to ensure clarity and contextual appropriateness before data collection.

In addition, demographic information was collected at the beginning of the survey, including gender, age, parental status and educational attainment.

Data were collected in September 2023 by a professional survey agency, which recruited respondents from its online panel with the aim of obtaining a sample broadly representative of the adult population in Slovakia.

### Data analysis

For data analysis, we employed Latent Class Analysis (LCA), which allows for the identification of hidden subgroups within a population (Weller et al., 2020). LCA utilises respondents’ answers as indicators to uncover the structure of latent classes, enabling the classification of individuals into distinct groups based on similarities in their response patterns (Nylund-Gibson & Choi, 2018; Wurpts & Geiser, 2014). In the context of this study, LCA facilitated the identification of segments within the Slovak population according to their attitudes toward COVID-19 vaccination.

Prior to conducting the LCA, responses were aggregated and averaged according to the respective HLQ scales, and items from the second questionnaire (Thaker & Cook, 2022) were conceptually grouped into five broader thematic domains: agreement with restrictions, communication, vaccination effectiveness, decision to vaccinate, and vaccination requirement. For the purposes of analysis, individual indicators representing these domains were analysed separately, resulting in eight variables reflecting vaccination-related attitudes and behaviours (see Table 4). These domains were selected to capture key attitudinal and behavioural aspects related to vaccination and public health compliance, reflecting both informational and motivational factors relevant to vaccine acceptance. This approach was adopted to reduce the number of variables, enhance the relevance of input data, and ensure efficient and interpretable data processing within the LCA framework.

The analysis was performed in R using the *poLCA* package (Linzer & Lewis, 2011). Models with two to five latent classes were estimated and compared using maximum likelihood estimation with robust standard errors. Missing data were handled through full information maximum likelihood (FIML).

To determine the optimal number of latent classes, models with two to five classes were compared based on several fit indices, including the Akaike Information Criterion (AIC), Bayesian Information Criterion (BIC), log-likelihood, and entropy values. Lower AIC and BIC values indicated a better model fit, while entropy values closer to 1.0 reflected greater classification accuracy and clearer separation between classes.

Following the identification of the optimal model, we conducted multinomial logistic regression analyses using the BCH approach to examine predictors of latent class membership while accounting for classification uncertainty.

#### Ethical aspects

The study was approved by the Ethics Committee of the Faculty of Social and Economic Sciences Comenius University Bratislava under reference number 161-56/2025. Participation was voluntary and based on informed consent, with respondents assured of anonymity and confidentiality. No identifying information was collected, and participants could withdraw from the study at any time without consequence. The research team took all necessary measures to ensure that data collection, analysis, and reporting were performed with integrity and respect for participants’ rights.

## Results

Models with two to five latent classes were estimated and compared based on model fit indices (Table 1). Both AIC and BIC values decreased progressively up to the four-class model, indicating improvement in fit. However, the five-class model showed only marginal improvement in AIC while slightly increasing the BIC value, suggesting potential overfitting.

**Table 1.** Characteristics of respondents by Latent Classes (*N* = 505).

Classes	Log-Likelihood	AIC	BIC	Entropy	Class1_n	Class2_n	Class3_n	Class4_n	Class5_n
2.	−12097.54	24529.07	25234.58	1.049	299	206	–	–	–
3	−11738.72	23979.43	25039.80	1.064	194	150	161	–	–
4	−11524.12	23718.24	25133.47	1.059	164	44	191	106	–
5	−11357.93	23553.87	25323.96	1.058	113	49	162	50	131

Note: *Values are presented as means with standard deviations or as frequencies with percentages.*

The three-class model demonstrated the most favourable balance between statistical fit and interpretability. Inspection of the four-class solution indicated that the additional class did not represent a substantively distinct profile but rather split one of the existing groups into two smaller segments with similar response patterns. The three-class solution also produced relatively balanced class sizes (*N* = 194, 150, and 161), supporting the interpretability of the model and indicating that the solution is not driven by small or poorly defined classes. This solution was therefore selected for subsequent analyses. Overall, the three-class model provided the most parsimonious and substantively meaningful representation of the observed response patterns.

Based on the selected three-class model, LCA identified three classes of respondents with a balanced age distribution (mean age of approximately 45 years across all classes). Differences between the classes emerged in gender composition, with men slightly predominating in Classes 2 and 3, whereas women were more prevalent in Class 1. In each class, approximately two-thirds of respondents had children. Educational attainment also differed across classes, with secondary and master’s education being most common. Descriptive characteristics of respondents by latent classes are presented in Table 2.

**Table 2.** Characteristics of respondents by Latent Classes (*N* = 505).

Characteristics	Class 1 (*N* = 194)	Class 2 (*N* = 150)	Class 3 (*N* = 161)
Age, M (SD)	45.14 (15.35)	45.00 (16.67)	45.03 (16.40)
Male, *n* (%)	76 (39%)	79 (53%)	89 (55%)
Female, *n* (%)	118 (61%)	71 (47%)	72 (45%)
With children, *n* (%)	125 (64%)	95 (63%)	107 (66%)
Without children, *n* (%)	69 (36%	55 (37%)	54 (34%)
Education—Primary, *n* (%)	9 (4%)	1 (1%)	2 (1%)
Education—Secondary, *n* (%)	116 (60%)	79 (53%)	78 (48%)
Education—Bachelor’s, *n* (%)	17 (9%)	11 (7%)	9 (6%)
Education—Master’s, *n* (%)	50 (26%)	51 (34%)	65 (41%)
Education—Doctoral, *n* (%)	2 (1%)	8 (5%)	7 (4%)

Note: *Values are presented as means with standard deviations or as frequencies with percentages.*

We then examined health literacy across the latent classes, measured across nine HLQ dimensions. Mean scores for the nine HLQ dimensions across latent classes are presented in Table 3. Overall, the results revealed relatively similar levels of health literacy between Classes 1 and 2, whereas Class 3 consistently reported higher scores across all dimensions. The most pronounced differences were observed in *Having sufficient information to manage health* (Class 1 = 3.40, Class 2 = 2.99, Class 3 = 3.94), *Ability to actively engage with healthcare providers* (Class 1 = 3.43, Class 2 = 3.05, Class 3 = 3.96), *Navigating the healthcare system* (Class 1 = 3.27, Class 2 = 2.88, Class 3 = 3.79), *Finding good health information* (Class 1 = 3.65, Class 2 = 3.22, Class 3 = 4.04), and *Understanding health information* (Class 1 = 3.69, Class 2 = 3.26, Class 3 = 4.11). In contrast, only minor differences were found in dimensions such as *Feeling understood and supported by healthcare providers* or *Appraisal of health information*. These findings indicate that respondents in Class 3 demonstrate a generally higher level of health literacy, while Classes 1 and 2 are characterised by medium scores with only small variations.

**Table 3.** Mean scores (and standard deviations) of HLQ dimensions across latent classes.

HLQ Dimension	Class 1 (*N* = 194)	Class 2 (*N* = 150)	Class 3 (*N* = 161)
HPS	M = 2.81 (0.57)	M = 2.78 (0.47)	M = 3.20 (0.51)
HIS	M = 3.40 (0.66)	M = 2.99 (0.52)	M = 3.94 (0.38)
AMH	M = 2.81 (0.57)	M = 2.71 (0.46)	M = 3.00 (0.56)
SS	M = 2.86 (0.52)	M = 2.76 (0.52)	M = 3.13 (0.54)
CA	M = 2.79 (0.53)	M = 2.81 (0.43)	M = 3.04 (0.52)
AE	M = 3.43 (0.74)	M = 3.05 (0.51)	M = 3.96 (0.43)
NHS	M = 3.27 (0.71)	M = 2.88 (0.57)	M = 3.79 (0.59)
FHI	M = 3.65 (0.63)	M = 3.22 (0.54)	M = 4.04 (0.45)
UHI	M = 3.69 (0.62)	M = 3.26 (0.62)	M = 4.11 (0.44)

Note: *Values are presented as mean (standard deviation) for each HLQ dimension. Higher scores indicate greater levels of the respective construct.*

In addition to health literacy, we further examined respondents’ perceptions and behaviours related to vaccination against COVID-19 across eight thematic areas. Mean scores for these variables are presented in Table 4.

**Table 4.** Mean scores (and standard deviations) of vaccination-related attitudes and behaviours across latent classes.

Dimension	Class 1 (*N* = 194)	Class 2 (*N* = 150)	Class 3 (*N* = 161)
Adherence to restrictions	M = 3.87 (1.24)	M = 2.57 (0.84)	M = 2.51 (1.04)
Communication	M = 5.00 (1.06)	M = 4.62 (0.83)	M = 3.34 (1.01)
Vaccination effectiveness	M = 1.77 (0.84)	M = 4.36 (1.12)	M = 4.84 (0.98)
Past vaccine refusal	M = 2.83 (2.04)	M = 4.96 (1.46)	M = 5.37 (1.32)
Vaccination requirement	M = 4.07 (1.01)	M = 2.05 (0.97)	M = 1.59 (0.76)
Self-reported vaccination	M = 1.71 (0.45)	M = 1.07 (0.26)	M = 1.04 (0.20)
Community protection	M = 3.76 (0.43)	M = 1.82 (0.81)	M = 1.69 (0.81)
Agreement with restrictions	M = 5.65 (0.77)	M = 3.60 (1.28)	M = 3.65 (1.41)

Note: *Values are presented as mean (standard deviation) for each dimension. Adherence to restrictions refers to compliance with preventive measures during the COVID-19 pandemic. Past vaccine refusal denotes attitudes toward vaccination prior to the COVID-19 pandemic. Self-reported vaccination indicates whether respondents themselves were vaccinated against COVID-19. Community protection reflects vaccination motivated by the desire to protect one’s community. Agreement with restrictions refers specifically to agreement with restrictions applied to unvaccinated individuals.*

Class 1 reported the highest *adherence to restrictions during the pandemic* (M = 3.87), indicating stronger compliance with preventive measures, as well as the highest *agreement with restrictions for the unvaccinated* (M = 5.65). They also expressed the highest level of trust in *communication* (M = 5.00). However, this group simultaneously demonstrated the lowest perceived *vaccination effectiveness* (M = 1.77) and the lowest level of *self-reported vaccination against COVID-19* (M = 1.71).

Classes 2 and 3, in contrast, exhibited substantially higher confidence in *vaccination effectiveness* (M = 4.36 and M = 4.84, respectively) and strong disagreement with *past vaccine refusal before COVID-19* (M = 4.96 and M = 5.37), suggesting greater vaccine acceptance. Nevertheless, both groups expressed weaker agreement with the *vaccination requirement* (Class 2: M = 2.05; Class 3: M = 1.59) and lower endorsement of *community protection as a reason for vaccination* (Class 2: M = 1.82; Class 3: M = 1.69).

Overall, these results suggest that Class 1 represents individuals who were highly compliant with public health restrictions but relatively sceptical toward vaccination. In contrast, Classes 2 and 3 reflect respondents who were more confident in the effectiveness and necessity of vaccination, while being less supportive of mandatory vaccination policies and less motivated by community-driven reasons for vaccination.

To formally examine which variables predict class membership while accounting for classification uncertainty, we conducted a multinomial logistic regression analysis using the BCH approach, with Class 2 specified as the reference category (Table 5).

**Table 5.** Multinomial logistic regression predicting latent class membership (reference category: Class2).

Variable	Class 3 vs 2 (OR [CI])	p	Class 1 vs 2 (OR [CI])	p
HPS	6.97 [1.23, 39.33]	0.028	1.76 [0.01, 229.42]	0.819
HIS	28.02 [6.25, 125.62]	<.001	2.05 [0.03, 128.39]	0.733
AMH	4.27 [0.91, 20.07]	0.066	0.53 [0.01, 25.83]	0.749
SS	3.99 [0.72, 22.05]	0.113	1.85 [0.06, 60.67]	0.730
CA	1 1.64 [0.29, 9.42]	0.578	0.05 [0.00, 12.75]	0.286
AE	2 1.22 [4.51, 99.72]	<.001	0.66 [0.01, 74.82]	0.864
NHS	7.88 [2.08, 29.81]	0.002	13.17 [0.18, 952.21]	0.238
FHI	11.18 [2.52, 49.64]	0.002	1.14 [0.05, 25.84]	0.934
UHI	14.60 [2.66, 80.09]	0.002	4.41 [0.12, 165.40]	0.423
Adherence	0.63 [0.31, 1.27]	0.196	4.99 [0.63, 39.43]	0.127
Communication	1.90 [0.81, 4.44]	0.140	0.08 [0.01, 1.02]	0.052
VaccineEff	1.80 [0.90, 3.59]	0.095	0.73 [0.21, 2.48]	0.613
PastRefusal	0.47 [0.16, 1.40]	0.177	17.42 [0.85, 357.96]	0.064
VaccReq	1.58 [0.04, 56.49]	0.802	132.84 [1.98, 8902.25]	0.023
SelfVac	0.94 [0.37, 2.40]	0.893	1315.37 [12.66, 136640.08]	0.002
CommunityProt	1.08 [0.60, 1.97]	0.789	5.86 [1.18, 29.20]	0.031
AgreeRestr	0.83 [0.37, 1.88]	0.661	1.51 [0.34, 6.76]	0.589

Note: *OR = odds ratio; CI = confidence interval. Values greater than 1 indicate higher likelihood of belonging to the target class relative to the reference class. Values less than 1 indicate lower likelihood. Boldface indicates statistically significant effects at p < .05. Variable names correspond to Table 4 and Table 3 dimensions.*

Higher levels of health literacy consistently predicted membership in Class 3 compared to Class 2. In particular, HLQ2 (OR = 28.02, 95% CI [6.25, 125.62], *p* < .001) and HLQ6 (OR = 21.22, 95% CI [4.51, 99.72], *p* < .001) showed strong positive associations. Several additional HLQ dimensions (HLQ1, HLQ7, HLQ8, HLQ9) were also significant predictors, indicating that Class 3 is characterised by generally higher levels of health literacy. In contrast, none of the health literacy variables significantly differentiated Class 1 from Class 2.

Regarding vaccination-related variables, membership in Class 1 (relative to Class 2) was significantly associated with CB10 (OR = 132.84, 95% CI [1.98, 8902.25], *p* = .023), CB13_A (OR = 1315.37, 95% CI [12.66, 136640.08], *p* = .002), and CB15 (OR = 5.86, 95% CI [1.18, 29.20], *p* = .031). No vaccination-related variables significantly predicted membership in Class 3 compared to Class 2.

Overall, these findings suggest that Class 3 is primarily distinguished by higher health literacy, whereas Class 1 differs from Class 2 mainly in vaccination-related attitudes rather than health literacy.

## Discussion

The present study aimed to explore patterns of vaccination-related attitudes and behaviours within the Slovak population, alongside differences in health literacy levels across latent classes. Using Latent Class Analysis (LCA), we identified three distinct groups that differ not only in their perceptions of COVID-19 vaccination but also in their general approach to public health measures and communication.

Overall, the findings revealed a complex relationship between trust in vaccination, adherence to restrictions, and health literacy. Respondents in Class 1 demonstrated the highest adherence to pandemic-related restrictions and trust in official communication but simultaneously expressed scepticism toward the effectiveness of COVID-19 vaccination and reported the lowest likelihood of being vaccinated. This combination of compliance with restrictive measures yet hesitancy toward vaccination may reflect a reliance on external regulation rather than intrinsic health motivation. In other words, adherence to public health rules may in some cases reflect behavioural compliance with institutional directives rather than the internalisation of pro-vaccination beliefs. Importantly, this pattern also suggests that vaccination attitudes cannot be explained solely by informational or cognitive capacities, but may also reflect differences in normative orientations toward rules and varying levels of institutional trust. In contrast, Classes 2 and 3 exhibited stronger belief in the effectiveness of vaccination, higher self-reported vaccination rates, and clear disagreement with past vaccine refusal. These groups also showed higher average levels of health literacy, particularly in the domains of navigating the healthcare system and understanding health information, indicating an association between higher health literacy and more positive vaccination attitudes. At the same time, the differences between Classes 2 and 3 suggest that they may not simply represent different points on a single continuum of vaccine confidence or literacy, but rather reflect distinct orientations toward vaccination and public health communication.

The observed segmentation aligns with previous findings by Thaker (2021a), who identified four distinct public groups based on vaccination beliefs—*vaccine enthusiasts*, *supporters*, *hesitant individuals*, and *sceptics*. In his later study conducted in Australia, Thaker et al. (2023) extended this typology by adding a fifth group, referred to as *vaccine socials*, representing individuals who were undecided but inclined to vaccinate primarily to protect themselves and others. However, in our study, three classes emerged, broadly corresponding to Thaker’s typology. Class 1 may be viewed as *restriction-oriented sceptics*—respondents who supported public health restrictions and trusted official communication but remained doubtful about the effectiveness of vaccination and reported low vaccination rates. This combination of high compliance with public health restrictions and scepticism toward vaccination represents a theoretically interesting pattern that has been noted in pandemic-related research. Rather than reflecting simple opposition to institutional authority, it may indicate a form of authority-based compliance in which individuals follow externally imposed rules while remaining cautious about specific medical technologies or interventions. This distinction highlights that behavioural compliance with public health measures does not necessarily imply a deeper internalisation of the beliefs or values underlying those measures. Class 2 could be characterised as *hesitant supporters*, displaying moderate confidence in vaccines and partial agreement with vaccination norms, which may reflect a more cautious or conditional acceptance of vaccination, while Class 3 resembles *vaccine enthusiasts*, expressing the strongest belief in vaccine effectiveness, highest vaccination rates, and the most positive perception of vaccination, suggesting a more firmly internalised pro-vaccination orientation rather than merely a higher level of confidence. Among these groups, the profile of restriction-oriented sceptics is particularly noteworthy because it combines high compliance with public health restrictions with low confidence in vaccination.

Several mechanisms may help explain the profile of restriction-oriented sceptics. First, compliance with restrictions may stem from risk aversion and a general preference for precautionary behaviour during uncertain public health crises. Individuals who perceive the pandemic as threatening may therefore support restrictive measures even if they remain uncertain about the safety or effectiveness of newly developed vaccines. Second, scepticism toward vaccination may reflect selective distrust directed specifically at biomedical or technological interventions rather than a broader rejection of institutional guidance. Finally, historical and sociocultural factors may also play a role. In post-communist societies such as Slovakia, long-standing ambivalence toward state authority can coexist with pragmatic compliance with regulations, producing a pattern in which individuals adhere to formal rules while maintaining sceptical attitudes toward specific policy measures or recommendations.

Importantly, this distinction highlights that behavioural compliance with public health measures does not necessarily imply a deeper internalisation of the beliefs or values underlying those measures. Integrating the concept of medical healthism—which frames vaccine attitudes as embedded in moralised discourses of personal responsibility, autonomy, and ‘doing health correctly’ (Kirbiš, 2023)—helps explain why individuals with comparable health literacy may still diverge in their vaccination decisions. In particular, restriction-oriented sceptics may comply with rules due to perceived external obligations while maintaining scepticism toward vaccines as part of a broader normative orientation shaped by trust, institutional experience, and personal health ideology.

Taken together, these findings suggest that vaccine hesitancy does not necessarily coincide with general non-compliance with public health measures, but may instead reflect more nuanced patterns of trust, risk perception, and institutional legitimacy within different population segments. In line with recent person-oriented research (Lamot et al., 2024), this pattern further supports the view that vaccine hesitancy is not a uniform construct but consists of distinct configurations of attitudes and beliefs rather than a single continuum.

These patterns also raise the question of whether differences in health literacy may help explain the observed variation across classes. Specifically, our multinomial logistic regression analyses (Table 5) showed that higher health literacy substantially increased the likelihood of belonging to Class 3 relative to Class 2, whereas membership in Class 1 was primarily predicted by vaccination-related attitudes rather than health literacy. This pattern confirms that Class 3 is characterised by generally higher literacy, while Class 1 differs mainly in scepticism toward vaccination, highlighting the distinct role of cognitive versus attitudinal factors in shaping vaccine-related behaviours. Our results thus extend Thaker’s framework (2021a) by suggesting that health literacy may represent an important differentiating factor across these groups. Individuals with higher levels of health literacy tended to report greater confidence in vaccination and a more autonomous, informed approach to health decisions. At the same time, health literacy should be understood as only one component within a broader constellation of factors shaping vaccination attitudes, alongside normative orientations toward collective rules and varying levels of institutional trust. Conversely, lower literacy was associated with persistent uncertainty and ambivalence, reinforcing dependence on external authority and restrictive measures rather than autonomous, informed decision-making.

These findings are consistent with prior evidence that vaccine acceptance is shaped by a complex interplay of cognitive, social, and institutional factors. Previous research has shown that (mis)trust in healthcare professionals and institutions significantly affects individuals’ willingness to vaccinate (Masaryk, 2016; Murphy et al., 2021; Raffetti et al., 2022; Roy et al., 2022; Zvoníček, 2019). The relatively high trust in official communication observed among the more sceptical class in our study may reflect a form of compliance driven by perceived authority rather than internalised conviction. Furthermore, educational attainment—often linked with higher health literacy—has been repeatedly associated with greater vaccine confidence (Fisher et al., 2020; Veldwijk et al., 2015), which corresponds to the higher literacy and vaccination rates observed in Class 3.

In line with studies highlighting the influence of social reference groups and belief in conspiracy theories (Masaryk, 2016; Murphy et al., 2021; Roy et al., 2022), our results also indicate that individuals with lower health literacy may be more susceptible to external social influences. This could help explain why some respondents, despite expressing general trust in restrictions, remained hesitant or refused vaccination altogether. Finally, the link between religious or ideological worldviews and vaccine scepticism observed in prior studies (Murphy et al., 2021; Troiano & Nardi, 2021) may provide an additional interpretive framework for understanding differences between the more vaccine-confident and vaccine-sceptical groups identified here.

These findings suggest that vaccination attitudes in Slovakia are shaped by competing social narratives—one grounded in institutional trust and scientific reasoning, and another rooted in experiential or socially shared scepticism. The co-existence of these narratives helps explain why certain individuals may comply with public health measures at the behavioural level while not fully internalising pro-vaccination beliefs at the attitudinal level. Such discrepancies may also reflect broader socio-historical influences. Post-communist countries, including Slovakia, have a well-documented tradition of mistrust toward official authorities and state institutions, a legacy stemming from decades of political control and limited transparency under communist regimes (Rose-Ackerman, 2001; Watier & Marková, 2004). From a broader comparative perspective, these findings may also inform discussions about vaccination attitudes in other low-trust democratic contexts, where institutional scepticism and reliance on informal information networks can shape how individuals interpret public health recommendations. This enduring scepticism is theorised as a mechanism influencing vaccination-related attitudes: lower institutional trust may reduce confidence in government-led health campaigns and increase reliance on informal sources when forming health-related decisions, helping to explain why some individuals remain hesitant despite complying with public health measures (Lášticová & Kende, 2023; Ramonaitė, 2023).

These insights have important implications for public health communication. Efforts to increase vaccination uptake should not solely focus on providing factual information but should also address the interpretive frameworks through which individuals make sense of that information. Strengthening health literacy—particularly in the areas of evaluating and applying health information—may support individuals’ capacity for more informed health decision-making. Communication strategies should therefore be adapted to different public segments: messages targeting vaccine sceptics should emphasise trust-building and personal relevance, while communication for the hesitant should focus on reducing uncertainty and improving access to credible information.

Finally, this study highlights the importance of considering both cognitive and social dimensions of vaccine acceptance. While health literacy supports informed evaluation, social representations shape the meaning individuals attach to vaccination. This underscores the need for targeted communication strategies that not only provide factual information but also foster individuals’ capacity to critically evaluate and apply health-related knowledge within their sociocultural context.

Despite these contributions, several limitations should be acknowledged. First, the data were collected through a self-administered questionnaire, which limited the possibility of clarifying respondents’ interpretations or exploring their reasoning in greater depth. Second, as the study relied on self-reported measures, the results may be influenced by social desirability bias or subjective interpretation of the items. Third, the cross-sectional design does not allow for causal inferences regarding the relationships between health literacy, attitudes, and vaccination behaviours.

Although the sample was designed to be demographically representative of the Slovak adult population, certain subgroups—such as individuals with strong anti-vaccination attitudes or those with limited internet access—may still be underrepresented. Finally, given the specific sociocultural context of Slovakia, characterised by relatively low institutional trust and vaccination rates, the generalisability of the findings to other countries should be made with caution.

## Conclusion

The present study provides an empirically grounded perspective on how Slovak adults differ in their perceptions of vaccination, adherence to restrictions, and health literacy. Using Latent Class Analysis, we identified three distinct groups that mirror the typologies described in previous international research, yet reflect the unique sociocultural context of Slovakia—a country marked by persistently low vaccination rates and declining public trust in institutions. At the same time, the findings may also contribute to broader discussions on vaccine hesitancy and public health communication in societies characterised by comparatively low levels of institutional trust.

Our findings indicate that health literacy is an important characteristic distinguishing population segments with differing vaccine-related beliefs and behaviours. Individuals with higher health literacy tended to demonstrate greater confidence in vaccination and a more autonomous, informed approach to health decisions, whereas those with lower literacy appeared to be more reliant on external authority and restrictive measures. The regression results further support this distinction, showing that Class 3 membership is strongly associated with higher health literacy, while Class 1 membership is mainly linked to vaccination-related scepticism rather than literacy levels. These findings underscore that interventions aiming to improve vaccine uptake may need to address both knowledge-based and attitudinal components. Overall, variations in health literacy correspond to distinct patterns of vaccination-related attitudes across the population.’

Beyond its theoretical contribution, the study offers practical implications for public health policy. Communication efforts should prioritise clarity, accessibility, and credibility while being tailored to the specific informational needs of different population segments. Interventions that combine education, transparent messaging, and community engagement may represent potential pathways for addressing informational barriers and supporting more informed vaccination decisions, while also contributing to the development of more sustainable public trust in health systems.

Future research should build on these findings by employing longitudinal or mixed-method designs to explore causal mechanisms and deepen understanding of how health literacy is related to trust, social influence, and institutional communication across time.

*The data that support the findings of this study are available from the corresponding author, upon reasonable request.*

## Data Availability

The data presented in this study are available on request from the corresponding author.
